# A focus Group Study of Medical Students’ Views of an Integrated Complementary and Alternative Medicine (CAM) Curriculum: Students Teaching Teachers

**DOI:** 10.3885/meo.2008.Res00252

**Published:** 2008-03-26

**Authors:** Désirée Lie, Johanna Shapiro, Sarah Pardee, Wadie Najm

**Affiliations:** Department of Family Medicine, University of California, Irvine, Orange, CA USA

**Keywords:** Curriculum assessment, Complementary and Alternative Medicine (CAM), focus groups, medical students, qualitative

## Abstract

**Background:**

Student views of new curricula can shape training outcomes. This qualitative study elicited student opinions of CAM instruction to examine and distill best strategies.

**Methods:**

49 second, third and fourth year students participated in focus groups using a predefined question route. Interviews were audio taped and transcribed.

**Results:**

Students successfully differentiated CAM curricula from other academic content and were supportive of a longitudinal integrated approach. They had positive disposition toward CAM use for themselves but this did not necessarily translate into patient recommendations. They agreed that goals of the CAM curriculum should center on awareness of patient use and evidence and information relevant to clinical practice. They advocated a case-based, hands-on, experiential strategy vs lectures. Students proposed greater institutional commitment to strengthen curricular effectiveness. The majority did not intend to practice CAM modalities but valued skills to assess them. Patient-centeredness was recognized. As training progressed, students exhibited a growing tendency to evaluate CAM efficacy, and therefore value, exclusively according to evidence.

**Conclusions:**

In-depth student input allowed examination of the effectiveness of a CAM curriculum, permitting improvement and assessment of program effectiveness.

The increasing popularity and use of complementary and alternative medicine (CAM) therapies and integrative medicine^1^–^5^ have stimulated new curricula.^6^ Many US health professions programs now offer courses containing CAM content.^7^,^8^ Students have shown positive attitudes to CAM training and recognized the need to have CAM therapeutic options available to patients.^9^,^10^,^11^ Recently, issues addressing the integration of CAM curricula into allopathic health professions training were highlighted in 9 articles in a 2007 supplemental issue of Academic Medicine^12^ summarizing the experiences of 20 US schools that received funding from the National Center for CAM^13^ to implement and evaluate such curricula. Among the lessons learned, was the importance of planned evaluation of learners and programs,^14^,^15^ with attention to nurturing student outlook and responding to students’ views and concerns. Because CAM is among a myriad of “hot” topics (e.g., domestic violence, nutrition) within existing curricula, its teaching has often been integrated into courses and clerkships or has occurred in electives rather than in the mainstream curriculum.^7^,^8^ There is an argument that CAM instruction belongs in the general professionalism curriculum^16^ and inquiring about CAM practices and beliefs should be part and parcel of every patient encounter, an approach also taken by educators incorporating cultural competence training.^17^–^20^

Information is emerging about student health professionals’ attitudes about CAM,^22^–^26^ but there is a paucity of literature describing student learning and student opinions of CAM curricula, in part due to the difficulties of evaluating programs longitudinally when curricula are ‘hidden’ in other experiences such as standardized patient (SP) cases, precepting practices, evidence-based medicine (EBM) instruction and community-based experiential learning. In addition, it is possible that students are not taught to distinguish among closely related topics when teaching occurs in the clinical setting, or they may view topics such as diversity, professionalism, CAM and EBM as so interconnected as to be indistinguishable.

To assess CAM curriculum program effectiveness, we conducted a focus group study of second, third and fourth year medical students in a medical school in which CAM teaching was introduced and integrated into existing courses within all four years of the curriculum. We had previously demonstrated as a baseline needs assessment an overall positive attitude toward CAM instruction in three consecutive cohorts of students using a validated survey instrument, the CAM Health Beliefs Questionnaire or CHBQ.^11^,^26^ However, the survey methodology that provided aggregate class mean scores for attitudes toward CAM practices did not permit examination of reasons for student attitudes toward CAM, toward the CAM curriculum itself and the impact of the curriculum on intended practice. Therefore, our purpose for this follow-up study was, first, to elicit detailed student-driven reflection on their curricular experiences and, second, to examine the effectiveness of our longitudinal curricular integration strategy with the overall goal of refining the integrated CAM curriculum. We hypothesized that there would be a range of opinions about the relevance to future practice and the efficacy of the CAM curriculum, in part associated with students’ attitudes toward and personal experience of CAM and their perceptions of institutional culture around CAM use and practice, and that the focus group process would allow links between the two to emerge.

Focus groups have been defined as a particular form of group interview intended to take advantage of group dynamics by stimulating conversation among participants.^27^ The guiding principle is that the psychological processes help people to identify, reflect on, and clarify their own views and attitudes.^28^ Qualitative methods in general, and focus groups in particular, are a useful approach when dealing with issues that involve differing opinions, needs, values, and perceptions, especially among groups that do not systematically exercise institutional power.^29^ They are increasingly used in evaluation of health services^30^ and have been applied to elicit the voice of medical students in relationship to various curricular issues.^31^,^32^ Focus groups can provide insights into those aspects of the medical curriculum that are not amenable to study using conventional methods, such as cultural sensitivity,^33^ ethical issues and the hidden curriculum^34^ and, more recently, CAM curricula.^35^ We selected the focus group as our methodology because of its ability to elicit group and individual responses, to derive information on ‘hidden agendas’, its practical utility, and the availability of institutional expertise with this research tool. The project received prior Institutional Review Board Approval.

## Methods

**Setting -** The study was conducted in one California medical school with a class size of 92 students annually. Class demographics have been stable over the previous 4 years, with 50% female, 45 to 50% white, 30 to 40% Asian and 10 to 15% other ethnicities. The curriculum in years 1 and 2 comprised basic science teaching in traditional topics and a longitudinal two-year organ-based Doctoring course consisting of standardized patient (SP) cases in year 1 followed by patient encounters in community preceptors’ practices in the second year, culminating in a clinical skills examination at the end of year 2. From 2000 to 2003, additions were progressively made in CAM teaching, to include, by 2004, the following components of required CAM teaching (see Table 1): 1) Patient panels led by faculty (two hours) in year 1, 2) a discussion of the book The Spirit Catches You and You Fall Down^36^ (two hours), 3) searching CAM databases in an EBM class (two hours), all within the Doctoring course, 4) massage therapy taught in the Anatomy course (two hours), and 5) acupuncture taught within the Physiology course (one hour). In the second year, students experienced CAM curricula by completing interviews in their preceptors’ practices and doing literature searches on evidence related to CAM to present in small group settings (two hours within the Doctoring course); teaching also occurred in the Pharmacology course (one hour) and in the Topics in Medicine course (one hour). In the third year, CAM teaching occurred in the Family Medicine clerkship as a small group seminar (two hours), within an Objective Structured Clinical Examination (OSCE) with feedback (one hour), in the Emergency Medicine rotation (one hour), the Psychiatry clerkship (two hours) and the Obstetrics and Gynecology clerkship (one hour). Other required curricular experiences that may also impact CAM learning included community visits to local *botanicas* where patients obtained herbal medicines and bioethics teaching within clerkships (Surgery, Obstetrics and Gynecology or OB-Gyn and Family Medicine) that included patient health beliefs and diversity teaching within their materials.

**Table 1:** Integrated CAM Curriculum Year 1–4, Courses and Hours University of California, Irvine School of Medicine

Year	Required Courses				Electives
**1**	*Anatomy*	*Physiology*	*Patient-Doctor Course*	*Neurosciences*	20 hour elective in PD courses (completed by 20% of class)
	Massage (2 hours)	Acupuncture (1 hour)	CAM (2 hours)	Stress Management	
			EBM (2 hours)	& Medication (1 hour)	
			Culture (2 hours)		
**2**	*Pharmacology*	*Topics in Medicine*	*Patient-Doctor Course*		
	Herbals (1 hour)	Manipulation (1.5 hours)	Interviewing (1 hour)		
			CAM (1 hour)		
			EBM (lhour)		
**3**	*Family Medicine clerkship* (6 hours)	*Pediatrics clerkship*	*Ob-Gyn clerkship*	*Emergency Medicine*	4 week CAM elective (completed by 10% of class)
	*Botanicas* visit	Cancer (1 hour)	Menopause (1 hour)	Herbals (1 hour)	
	GMV		*Surgery*	*Psychiatry*	
	Reflective Practice		Bioethics/CAM (1 hour)	Relationships	
	OSCE			& Stress (1 hour)	
**4**					4 week CAM elective (10% of class)

Index of abbreviations: PD = Patient-Doctor course, EBM = evidence-based medicine, GMV= group medical visits, OSCE = Objective Structured Clinical Examination, Ob-Gyn = Obstetrics and Gynecology

In addition to required experiences, students had the option of participating in a 15 hour elective in year 1 and a four-week community practice-based elective with CAM practitioners in year 4. Ten to thirty percent of students took part in these experiences in any given year.

**Subjects and recruitment** - 47 students participated in 8 focus groups that targeted 2^nd^ (MS2), 3^rd^ (MS3), and 4^th^ (MS4) years of training; 2 third year female students were interviewed together as a “mini-group” for a total of 49 students. Participant breakdown by year and gender is summarized in Table 2. Recruitment consisted of two group emails sent to each class, flyers placed in the mailboxes of MS2s and 3s, flyers handed out in person by a research assistant after a class (MS2s only), and informal, word-of-mouth recruitment of peers by class leaders during required classes. Incentives to participate included a free lunch and a $20 gift certificate to the campus bookstore or a $25 book of the student's choice. All students shared a common background as a result of having participated in the required CAM curriculum. To ensure a wide range of opinions, efforts were made to recruit students who had diverse attitudes toward CAM use and the curriculum by stating in the recruitment announcements that ‘all points of view are welcome and encouraged’. Student responses were confidential, and the focus groups were conducted by staff and faculty not associated with their evaluation. No student who responded to the invitation was refused participation.

**Table 2:** Focus Group Participants by Year and Gender University of California, Irvine School of Medicine

	Group	Year	Male	Female	Total
	1	2	1	5	6
	2	2	3	4	7
	3	2	3	4	7
	4	2	1	3	4
	5	3	4	1	5
	6 (mini)	3	0	2	2
	7	4	5	0	5
	8	3	2	5	7
	9	3	2	4	6
**Total**	9	–	21	28	49

All focus groups were audio-taped with participants’ permission. Audio-tapes were later transcribed in their entirety by a research assistant. In addition, the research assistant took field notes during the groups, including observations of group process, and recorded keywords, sentence fragments, and summaries of basic ideas/concepts. The research team reviewed all these sources in formulating conclusions. The researchers consisted of two family physicians, both with expertise in cultural competence and CAM curriculum development,^38^ a psychologist with experience in conducting focus groups and analyzing focus group data, particularly with reference to cross-cultural educational issues,^38^,^39^,^40^ and a research assistant.

**Figure 1:**
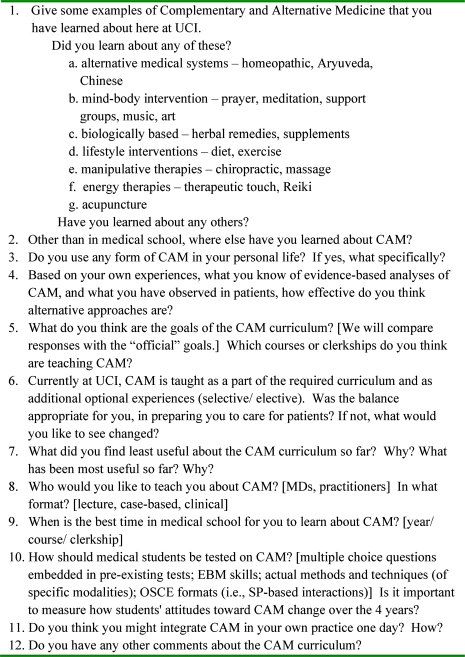
Question Route for CAM Focus Groups University of California, Irvine School of Medicine

**Focus group structure** - The researcher (JS) with extensive previous focus group experience examining cultural issues relevant to medical education developed a preliminary version of the focus group question schedule. A second researcher (DL) with expertise in CAM and curriculum development, a third researcher, a CAM practitioner and educator (WN) with curricular expertise, and the research assistant reviewed and modified this question route (see figure 1). The group met 3 times to establish consensus on the document. The question route was adhered to for each group. All groups conformed to standard focus group methodology, specifically establishing an informal, conversational environment, urging participants to express opinions at variance with others in the group, and encouraging participation of quiet group members.^36^ The focus groups were conducted in a small classroom while students were eating lunch. The length of the discussion ranged from 1 to 1.5 hours. An audit trail was established through audio-tapes and transcripts, two sets of field notes, and written interpretations of the groups by each researcher.

**Data analysis** - Every member of the analysis unit (JS, DL, WN) reviewed each group transcript separately and noted key words, phrases, and major themes, both in response to the specific questions asked and as they spontaneously emerged from student comments.^30^,^41^ Words and phrases related to the same content were grouped together and preliminary subcategories were formed; these were subsequently grouped into categories and themes.^42^,^43^ Each researcher listed important concepts for each set of focus group transcripts and then counted its occurrence. Researchers met face-to-face twice, discussed their individual interpretations of the group sessions, and identified similarities and points of disagreement. JS then integrated the topics identified and counted by the individual researchers into a comprehensive summary, reflecting all points of view across and within the focus groups analyzed. We used criteria of frequency (number of times mentioned in transcript), extensivity (amount of detail recorded), and intensity (language and expressions used by students) in evaluating the data. We compared student responses both across group and by year of training. Our aim was to detect important patterns and concepts that persisted across groups while also incorporating differences among groups, outlier positions, and contradictory data.

Data triangulation was achieved through multiple data sources. Investigator triangulation occurred because of the incorporation of different disciplinary perspectives into the data analysis. Member checking was conducted at the end of each focus group by checking our perceptions of the main points of the session with participants.^43^

## Findings

Overall, we concluded that we elicited a broad range of student opinions about CAM. After each group, the lead facilitator (JS) and the research assistant (SP) noted the presence of comments representing skepticism, enthusiastic endorsement, and neutrality (open-mindedness). In addition, post-hoc review of the transcripts confirmed that opinions of students regarding the CAM curriculum spanned the spectrum from highly positive to skeptical in most groups. The views tended to be similar within preclinical and clinical student groups. We were able to achieve theoretical saturation (that is, repetition of themes) of the data in both preclinical and clinical years. Whenever student perceptions diverged consistently by year, these findings are reported separately.

**CAM content in the curriculum** - Both preclinical and clinical students reported curricular exposure to information about acupuncture, diet and exercise, herbal remedies, and spirituality (meditation and prayer). Art and music therapy and cupping were consistently mentioned in the pediatric clerkship, and moxibustion on the OB-Gyn clerkship. Students reported rare or no exposure to chiropractic, manipulation, energy therapies, massage, aryuveda, and homeopathic remedies. Predictably, clinical students mentioned exposure to a greater number of modalities.

**Methods of presentation of CAM material** - Almost all second and third year students mentioned a particular evidence-based lecture on the cardiovascular benefits of acupuncture and that acupuncture was integrated into physiology lectures. They were also aware of a CAM first year elective and of CAM being integrated into a required first year standardized patient interview. Students repeatedly mentioned integration of CAM topics on the family medicine third-year clerkship. Some students also mentioned a CAM website, a CAM interest group, and a CAM weekend conference. Efforts to include CAM on other required clerkships such as psychiatry, OB-Gyn, and medicine and other CAM-related lectures were infrequently mentioned.

**Other sources for learning about CAM** - All students frequently reported learning about CAM through personal experience from family (especially grandparents and parents) and also self-initiated personal experience and friends. Other less frequently mentioned sources of information included the media and CAM-related websites. Clinical students sometimes mentioned learning about CAM from patients. The large majority of students from all years had some or a fair amount of exposure/experience with CAM. “My parents are Korean so they do a lot of alternative medicine. They do all the acupuncture; they do all the herbal medicines.” (MS3) “I get acupuncture every once in awhile and that cupping thing. And I drink like this herbal tea…but it's really nasty-tasting.” (MS2)

**Personal use of CAM** - Both second year and third or fourth year students tended to mention having tried such CAM modalities as herbals/teas, massage, chiropractic, exercise, and various nutritional interventions. Occasionally students were personally familiar with cupping, homeopathic or Chinese medicine, acupuncture, yoga, and meditation. Students never mentioned going to healers, homeopathy, or energy healing. “My mom is Indian and has a million concoctions and she throws them in milk or something gross and I have to drink it. So I do that all the time.” (MS3) “I used to use prayer to stay healthy. Sometimes I do massage, I do chiropractic. I've also gone to the flax and fish oil guy.” (MS2)

**Belief in CAM efficacy** - Among second year students, the most widely endorsed response was that CAM efficacy is highly variable, depending on the situation, the individual, and the type of modality being considered. Acupuncture was generally perceived as selectively effective for some conditions. There was more ambivalence about herbals. A minority across all years felt CAM was not at all effective. The majority of students were open to being persuaded of efficacy, and many felt positive about certain practices (such as acupuncture), but not others. Those who were open often recounted strongly positive personal anecdotes. A few, however, had become more skeptical about CAM because they believed that CAM was ‘not research-based’. “We're all about statistics and p-values, and all that other stuff. In order to use it in a western society, any type of eastern whatever… any type of CAM, I think you have to standardize it to the system that we're used to. Otherwise it's not really going to be valid” (MS2).

Clinical students expressed a wide range of opinion in terms of perceived efficacy of CAM. Some students adopted the position that if people believed in it, it generally does some good. Some argued that what we consider CAM is equal to western medicine but has not been validated by western norms. However, on the negative side, students expressed the concern that CAM is not necessarily benign, but we know very little about it. These students worried that people use CAM uncritically and that much more evidence is needed. “I don't know whether if it's actually the treatment that helps or just the placebo effect. But I think it's helpful.” (MS2) “I think what happens is that it works so well for some people, it becomes evidence to other people that it works. Where really it's not evidence, it's a story.” (MS2)

**What constitutes evidence of efficacy** - Discussing the issue of efficacy led us to ask about what kind of “evidence” was convincing to students. In the second year, there were some hard-liners: “If it's EBM-proven, then it transforms into real medicine.” Others put stock in “3,000 years of history,” for example, in the case of Chinese medicine. Testimonials of individual patients or friends and family seemed very compelling to preclinical students. They also expressed the view that patients “don't need” evidence if CAM is part of their culture. Some students were also willing to accept the “placebo effect” if the product had a positive outcome for the patient: “..…if it is something that's already in place in a patient's environment, you don't really need the evidence.” (MS2) “If (sic) this is the stuff they always do, and they're positive about it, I think that that makes it work better for them.” (MS3) “I think just for the placebo effect, it's very effective.” (MS3) “So you hear stories like that and it's really powerful, more so than any trial or study could be.” (MS3)

Among third and fourth year students, many, especially 4th year students, wanted “proof”, defined as clinical trials. This need for evidence was much stronger than among the second years. A few accepted the “proof” of historicity; another handful was influenced by personal anecdote. Some students suggested that, as an academic community, we need to be more open to creative ways of assessing efficacy other than the randomized clinical trial. On the whole, while open to the potential efficacy of CAM, these students were frustrated that there is not a lot of research being conducted and were focused on the potential risk of harm to patients in the absence of evidence. “We're not an alternative, a naturopathic school. We can't just talk… the way it's presented needs to be in a scientific manner … the way it's presented is too touchy-feely for me. It's too, ‘This is wonderful.”… where is the evidence?” (MS3) “…if I want to believe in something there has to be good evidence-based, statistical significance to what people are using…” (MS3)

Several students in both preclinical and clinical groups, although more so in the second year groups, discussed that evidence-based medicine (EBM) as a gold standard for efficacy might have limitations. Some regarded EBM as Truth with a capital t, but others recognized it as a culture-bound phenomenon. Third and fourth years were more intent on “wanting to know the science” behind CAM if it is presented in the curriculum (its mechanisms of action, pathways, EBM-proven efficacy). “…if you can incorporate CAM… and have those numbers to back it up, have some kind of evidence, because it's one thing to just say, ‘Well, I've heard that there's something called this, people take it when they're experiencing this’ but if you can back that up with numbers, it speaks a lot more…” (MS2)

**Goals of CAM curriculum** - Most students felt that the primary goal of the curriculum was *awareness*, to make students aware of CAM's existence: “It's out there and your patient might use it.” Both preclinical and clinical students frequently mentioned that the purpose of the curriculum was simply to get them to ask their patients about CAM: “…you need to be able to recognize that it exists and to be able to ask about it.” (MS3)

A smaller number of students mentioned goals of relating to patients using CAM practices with sensitivity and respect, transmission of (very limited) knowledge about CAM, influencing attitudes in a positive direction, and giving students tools to learn more. Particularly in the clinical years, students emphasized that the goal was not to learn to practice CAM or to know about all modalities. “The goal is to increase awareness of the treatments patients might be using. Awareness includes acceptance. Be wary of potential harms of CAM and also aware of the benefits.” (MS3)

A handful of students in both preclinical and clinical years had a skeptical view of the curriculum goals, labeling it as “just going through the motions” with “no real commitment”. These students wanted a more rigorous, in-depth curriculum. On the other hand, several students expressed the view that allopathic schools should not be in the business of educating doctors to “practice” CAM and that most allopathic physicians don't want to be “CAM practitioners.”

**Attitudes toward CAM** - Some students perceived one of the goals of the CAM curriculum to be improving students’ attitudes toward CAM. However, the majority believed CAM was presented in an “objective,” “scientific” way, not leaning toward either excessive endorsement or negativity, and they liked this approach. Fourth years were more likely than students in other years to say that participating in the curriculum made them feel more comfortable with CAM and about recommending it to their patients.

**Least useful aspects of CAM curriculum** - Opinions were divided among second year students, with some stating lectures were not memorable, while others thought lectures presented useful, “scientific” information. A standardized patient interview integrating CAM was judged by some students as too challenging and insufficiently educational, while others thought it served its purpose of preparing students for patients who use CAM. Exercises to interview and then discuss actual patients using CAM were also evaluated as not useful because of the difficulty in finding such patients. Preclinical students also requested more hands-on exposure: “I think you always learn a little bit more when you do something that's more interactive, more hands on. I would have liked to have more of an experience with various types of alternative medicine.” (MS2)

Clinical students complained that the curriculum provided inadequate understanding of what herbal remedies are for and how each one is used; “diet and exercise” prescriptions were encouraged without any meaningful understanding of nutrition or how to effectively counsel about weight loss; reflective discussion groups often seemed redundant; material was superficial and not well connected to clinical practice; written assignments seemed like busywork; and the curriculum overall was repetitive. A few clinical students noted that there was a prevalent feeling among most (other) students that CAM was just a “nuisance.” Overall, students preferred cases and demonstrations to lectures. “I feel like there's a discontinuity between teaching us what it is versus us actually being able to help our patients achieve what they want with it.” (MS3) “They let me go in and watch when they were doing it and the guy that was doing it explained it to me what he was doing and why and that was really helpful.” (MS2) “I think I learned what but I didn't learn how to transition that into practicing it or utilizing it in a practice setting.” (MS3)

**Most useful aspects of CAM curriculum** - Students mentioned experiential aspects and direct exposure, as well as certain reading materials and lectures. A few students noted that the mere presence of CAM in the curriculum was useful because it sent an important message about its relevance in medical education. Fourth year students were more likely than any other year of students to do their own PubMed or other database searches.

**Suggestions for improving CAM curriculum** - The most commonly mentioned suggestion among second year students was the need for a systematic overview of CAM information. Specifically, they wanted a single lecture providing a comprehensive overview of common (top 10) CAM practices, including definitions, examples, illustrative pictures, an identification of the most common uses of these practices, RCT-evidence of efficacy, and information about dangerous drug interactions and general harm: “..it would be more beneficial to me if I know what were the top ten supplements, the top ten maneuvers, and really more of the efficacy.” (MS3) However, a few students dissented, pointing out that “top 10” might vary from community to community or simply change, and argued that the curriculum shouldn't focus so much on content as on skills (how to search, for example).

By contrast, students in the clinical years stressed getting more practical training, including hands-on training, linking content information to clinical practice, and expanding field trips and community experiences that included observation of and interaction with practitioners and patients. They also suggested more training in how to integrate CAM with western medicine and specifically how to advise patients. Some students would have preferred more “useful” CAM, such as in-depth nutritional education as opposed to “far-out” CAM like aryuvedic because physicians make recommendations about these with most patients without being able to say more than “watch your diet,” or “get more exercise.” Many students also advocated for better content and better quality, not just “random smattering of very cursory lectures,” and a more useful and more efficient curriculum. Often, this seemed to be more teaching from scientific perspective, citing studies and evidence.

Other suggestions included a CAM Day to expose students in an experiential way, CAM “tag-alongs”, list of community CAM resources, panels of patients and practitioners, opportunities to do research, and incorporating CAM into existing EBM assignments. Most of these ideas came from second year students.

**Structure of CAM curriculum** - Required vs. elective: Students across all four years were reluctant to endorse more required CAM curriculum; most also did not see the need for additional electives, although a few did; a few thought it would be nice to have more optional CAM experiences. Among second year students, there was some sentiment that CAM should not be required at all because of resistance among their peers to attending lectures for any material not included on the US Medical Licensing Examination: “…we all focus on passing the boards. And anything that's not the exam… we don't really care about. It's so sad.” (MS2)

**Placement in curriculum** - Second year students sometimes suggested that CAM be required in 3rd year so long as it didn't supplant anything “more important”. Clinical students tended to say their years were already too full for more CAM, but agreed that the 3rd/4th years were the best place for CAM because of the greater potential for clinical applications. Both preclinical and clinical students agreed CAM should be present in 1st year, but in a very limited way, perhaps as one overview lecture; in the 2nd year, it could be linked to specific diseases and conditions. In the clinical years, the large majority of CAM curriculum occurred during the family medicine clerkship, and students agreed this was an ideal placement for the material.

**Curricular integration** - Students in both groups expressed the opinion that integration of CAM material in existing courses like Pharmacology, Topics in Medicine, Pathophysiology, and Biochemistry was desirable. Students wanted continuity and consistency: if they receive EBM validation of palmetto's efficacy in a family medicine CAM presentation, they would like the urologist on their urology rotation to be familiar with its use as well. Students differed about the extent to which such integration already existed. Students in the 3^rd^ and 4th years emphasized that there wouldn't be so much resistance if CAM were presented as part of traditional courses, whether basic science curriculum or clinical clerkships.

**CAM instructors** - Second year students were divided between thinking physicians with CAM expertise would make the best teachers versus actual CAM practitioners. Clinical students tended to favor physicians who knew about CAM. They were skeptical of both CAM practitioners and patient testimonials.

**Examinations and written assignments in CAM curriculum** - The majority of students agreed that CAM was hard to test. Many believed that CAM should not be tested at all or that it should not be tested unless it could be presented factually. Most clinical students expressed a favorable opinion about CAM testing in an OSCE format. Written assignments about CAM were regarded with even less favor than testing.

**Intentions to use CAM in students’ own practice** - Most second year students were open to the idea of making CAM-related referrals, but most did not intend to practice CAM themselves. Several students stated that they wouldn't recommend CAM as first line treatment, but they wouldn't object to their patients using a CAM modality so long as it wasn't harmful. A minority of students took the position that they would not use CAM in any way unless they learned more about it, especially from an evidence-based perspective. A handful of students intended to incorporate CAM into their practices: “I may not personally do acupuncture on my patients but if somebody wants to talk about it, I'll talk about it, and I will probably recommend vitamins and supplements. It's pretty much guaranteed that I'm going to involve nutrition, exercise, and spirituality.” (MS2) “…I won't tell them not to do it. As long as there's no harm to them from the herbal (sic) medication.” (MS3).

Clinical students expressed more reservations. On the whole, they were willing to learn more about CAM, but they wanted to see EBM evidence about benefit and harm, otherwise they would not recommend it. They frequently mentioned wanting “proof.” Others were concerned that CAM could be exploitive of patients in terms of unnecessary expenditures and even outright quackery. Similar to second year students, even students who were open to CAM stressed it should be seen as supplementary, not alternative: western medicine should be tried first and only if it didn't work should patients then turn to CAM.

The majority of clinical students assumed a *reactive* position regarding the role of CAM in their future practices: if patients want to use it and if there's no harm, they wouldn't recommend against it. A handful of students anticipated a more *proactive* role in relation to CAM, describing multidisciplinary practices that would incorporate an acupuncturist, naturopath, nutritionist, and massage. Overall the most common attitude was one of limited openness to some level of incorporation. They favored the idea of CAM presence in their practices in principle, but didn't feel they knew enough to take more than a passive stance regarding usage: “As long as I know it's safe, it won't hurt them; it's a natural product, that's fine. If it works or not, we'll leave it up to them.” (MS3)

## Discussion

Overall, the majority of medical students valued the CAM curriculum and expressed appreciation that it was represented in the larger teaching program. This finding has been verified by findings of the 2007 AAMC Graduation Questionnaire, an exit survey completed by the entire graduating class and compared with other US and Canadian medical schools.^44^ Students from our school were much more likely to identify curricular coverage of CAM as ‘adequate’ (vs. inadequate or excessive), at a rate of 86% compared with the national average of 76% (GQ Question #11^44^). Our students were also more likely to be comfortable in assessing health practices of patients using CAM compared with other school's respondents (79% agree to strongly agree for our students vs. 72% for all schools, GQ question #15). Students wanted a more serious, in-depth training in CAM, including integration in both basic science and clinical clerkships and faculty who were competent and comfortable to support the instruction of CAM experts. Clinical students who held a favorable view of CAM also wanted to see more “CAM-in-action”, clear and practical links between theory and praxis, as well as more experiential training. We identified an encouraging trend from second year students, who showed a tendency to dismiss or minimize curriculum not relevant to the US Medical Licensing Examinations, to the fourth year students who finally were beginning to search for CAM-related evidence themselves, and discuss and recommend CAM with patients based on published evidence.

Based on research assistant notes and facilitator observations, we believe students were comfortable and sincere in the sessions. They expressed themselves with vigor and passion and seemed to feel their opinions were taken seriously. Almost every group presented a significant diversity of opinions, both positive and negative, about the CAM curriculum.

Thus, as has been shown in other focus group-based research about CAM in particular^35^ and cultural sensitivity in general,^43^ many of our learners expressed an interest in this area yet felt inadequately prepared by their training to address it. Reflecting the Astin, Goaddard, & Forsys findings,^35^ our study identified similar barriers to student comfort with CAM, including lack of sufficient curricular time and a “larger cultural ethos” that was ambivalent about complementary approaches. However, the picture that emerged from our research is more complex than students’ simply wanting more exposure to CAM. Rather, we believe many students in our focus groups had assimilated the larger institutional culture's attitude of tolerance without full acceptance of CAM, and this was expressed in several negative ways.

We noted the tendency of some students to demean the content and process of the CAM curriculum. Students criticized the curriculum as repetitive and poorly organized. Phrases like “more systematic,” “more structured,” and “less anecdotal” seemed to be code words for “more scientific.” Along similar lines, students often recommended ideas, such as CAM web-site and resource links, or information about risks and adverse effects that were already provided in the curriculum, suggesting that they did not always pay careful attention to what they were being taught. Disturbingly, a small number of students admitted “making up” information to satisfy CAM assignments and justified this behavior by the triviality of the CAM curriculum as a whole. Many students also wondered whether CAM deserves serious consideration in the curriculum because there was insufficient “gold standard” (i.e., EBM) data to support its usage. Further, students became progressively narrower in what constituted “proof” of efficacy as they advanced through the educational system. Some questioned whether CAM belonged at all in the curriculum of an allopathic medical school.

These behaviors suggest that the perceived low institutional status of CAM affects the perceptions of certain students, permitting them to regard CAM as unimportant compared to the rest of the curriculum. Ironically, given this critical perception of the CAM curriculum we identified in our focus groups, students overall had a high level of exposure to some form of CAM. For the majority, their familiarity came from parents or grandparents who were 1st or 2nd generation immigrants and regularly recommended CAM practices to them. This familial link produced an intriguing personal/professional conflict. Students seemed willing to use CAM on themselves without strict evidence but were reluctant to recommend it to patients. This suggests students may apply one set of values in their personal lives but another in their professional lives. It raises the question of whether students are applying a higher standard of care to their patients or whether they have concluded that in order to become physicians they must abandon other potential standards of evidence as soft and unscientific. Overall, the principle of nonmaleficence appeared to dominate students’ responses about the role of CAM in their own future practice.

**Figure 2:**
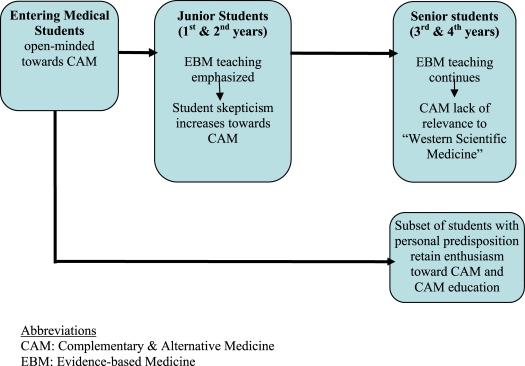
Theoretical Model Generated by Focus Group Findings University of California, Irvine, School of Medicine

Our research suggests the following theoretical model for understanding the complex relationship between CAM education and the overall process of medical education. Most students reported bringing an open mind to learning about CAM modalities and interventions. On the whole, they were happy that it is part of the curriculum. If anything, they wished to have more in-depth exposure and training. But – and this is key – increasingly they wanted this exposure to be presented in scientific terms as they have been taught to understand them, i.e., evidence-based medicine. While students in all years supported CAM as part of an integrated curriculum in the pre-clinical years, this was because integration would make CAM appear more “scientific.” In the clinical years, students struggled to find a connection between clinical scientific practice and CAM and hoped integration would fill the gap. Information about CAM that did not conform to the bioscientific model was regarded as anecdotal and soft. A subset of students appeared to retain their enthusiasm for CAM, and continuing to apply broader standards of evaluation (i.e., historical validation and personal stories) to criteria of efficacy. This model is represented in Figure 2. The model generated several testable hypotheses, including the following: 1) Students’ desire for CAM efficacy to be established exclusively through RCT evidence increases as they move through the curriculum; 2) a subset of students who have had positive personal and familial experiences with CAM and/or who have a broader philosophical definition of “efficacy” retain a more favorable attitude to CAM practices throughout their medical education.

Strengths of our study include its in-depth approach to a broad spectrum of students by level and student opinion, the diverse perspectives of the researchers, and their expertise in this methodology. Our approach also permitted a cross-sectional and comprehensive look across 4 years of curriculum, an approach that can be generalized to assessment of other similarly presented curricula. The main limitation of the study is that it was conducted at one institution with its own unique integrated CAM curriculum, institutional culture and student demographics, and the particular findings may not generalize to other settings. Nonetheless, general principles emerged that inform curricular development and assessment of CAM instruction that can guide the process of curricular integration of not only CAM but other similar content.

## Conclusion

In summary, our focus group findings from 3 different classes of students at one school confirmed the positive attitudes toward CAM use previously demonstrated with the survey approach^11^ and allowed deeper examination of the extent of CAM use for themselves vs. their future patients. Students were able to clearly identify CAM curricula as independent from other curricula and generally recalled specific teaching and learning throughout the 4 years of training. As training progressed, students demonstrated both greater tolerance of patients’ beliefs in CAM effectiveness in the face of inadequate research evidence and also developed greater skepticism toward some aspects of CAM, an ambivalence expressed in a general outlook of “first do no harm” for their future practice.
